# Prognostic and Therapeutic Potential of Nuclear Receptors in Head and Neck Squamous Cell Carcinomas

**DOI:** 10.1155/2009/349205

**Published:** 2009-09-24

**Authors:** Shirley K. Knauer

**Affiliations:** Department of Otorhinolaryngology, University of Mainz, Langenbeckstrasse 1, 55101 Mainz, Germany

## Abstract

Head and neck squamous cell carcinomas are among the most common neoplasms worldwide and characterized by local tumor aggressiveness, high rate of early recurrences, development of metastasis, and second primary cancers. Despite modern therapeutic strategies and sophisticated surgical management, overall survival-rates remained largely unchanged over the last decades. Thus, the need for novel treatment options for this tumor entity is undeniable. A key event in carcinogenesis is the uncontrolled modulation of genetic programs. Nuclear receptors belong to a large superfamily of transcription factors implicated in a broad spectrum of physiological and pathophysiological processes, including cancer. Several nuclear receptors have also been associated with head and neck cancer. This review will summarize their mode of action, prognostic/therapeutic relevance, as well as preclinical and clinical studies currently targeting nuclear receptors in this tumor entity.

## 1. Introduction

**Figure 1:**
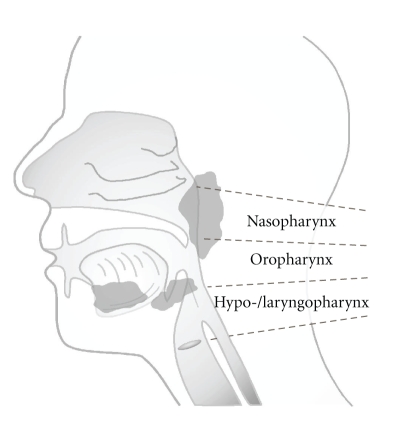
*Schematic anatomy of the head and neck region.* Head and neck cancer includes different types of malignancies that can develop in the mouth, nose and throat.

Most malignancies of the upper aerodigestive tract (Figure 1), comprising the naso-, oro-, hypo-, and laryngopharynx, are squamous cell carcinomas. Head and neck squamous cell carcinomas (HNSCCs) are the primary tumor type in head and neck cancer (HNC), characterized by local tumor aggressiveness, high rate of early recurrences, metastasis, and development of second primary tumors, which are the major cause of morbidity and mortality in HNSCC (details in [1–4]). More than 90% of HNC cases are induced by chronic exposure to carcinogens enclosed in all forms of tobacco, synergized by heavy alcohol consumptions and poor diet (see [5, 6]). It is estimated that about 5%–10% of suspicious lesions arising in the mucous membranes of the mouth, pharynx, and larynx undergo malignant transformation. Cure rates of early disease (stage I and II) range between 70% and 80%, and chemoprevention strategies seem promising to control potentially malignant oral lesions (reviewed in [1–3]). However, long-term survival rates, especially for advanced HNC, have not improved significantly over the last decades. Despite modern therapeutic strategies and sophisticated surgical management of the tumor, the estimated five-year survival rate for advanced disease (30%–40%) remains poor ([1–3] and references therein). Currently, rational therapeutic strategies targeting growth factor receptors by specific antibodies or kinase inhibitors have gained increasing clinical relevance in particular for the treatment of locally advanced cancer with the intent of preserving speech and swallowing (see [1–3]). Thus, developing new therapeutic strategies and defining novel target proteins for the treatment of advanced HNC is of particular importance.

In this respect, nuclear receptors (NRs) are transcription factors implicated in cancer development and are recently attracting major interest as therapeutic targets (see [7, 8]). As NRs modulate cell proliferation, apoptosis, invasion, and migration, clearly representing hallmarks of cancer cells, several highly successful cancer drugs target this receptor family [8–11]. Since several NRs have been shown to be expressed also in head and neck cancer cells, NRs are most likely also contributing to HNSCC development and progression [12, 13].

NRs belong to a large superfamily of transcription factors and based on sequence comparison are currently classified into seven subfamilies (Table 1). These transcription factors are able to modulate transcription of a variety of target genes by several distinct mechanisms, including both transcriptional activation and repression [7, 8, 14, 15]. Transcriptional regulation can either be ligand-dependent or -independent, genomic or nongenomic, allowing NRs to mediate gene repression or its release, gene activation, or gene *trans-*repression (details in [7, 8, 16]). In particular, the large group of so-called orphan nuclear receptors, for which natural ligands are still unknown, do not exist at all (true orphans), or have only recently been identified (adopted orphans) is adding additional complexity to the field (Table 1) ([8, 17], and references within).

**Figure 2:**
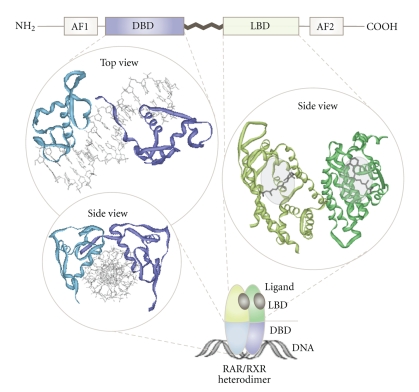
*Domain organization and structural binding modes of NRs.* Upper panel: NRs are composed of an N-terminal regulatory domain (activation function 1 = AF1), followed by a DNA-binding domain (DBD), a ligand-binding domain (LBD), and a C-terminal domain (activation function 2 = AF2). Left panel: 3D model illustrating how the DBDs of the RAR/RXR heterodimer (PDB 1DSZ) interact with their target DNA-sequence. Right panel: solid ribbon representation illustrating the LBD of the RAR/RXR heterodimer (PDB 1DKF) complexed with the ligands 9-*cis*-RA for RXR (PDB 3LBD) and ATRA for RAR (PDB 2LBD). PDB files are taken from the RCBS Protein Data Bank (http://www.pdb.org/).

**Figure 3:**
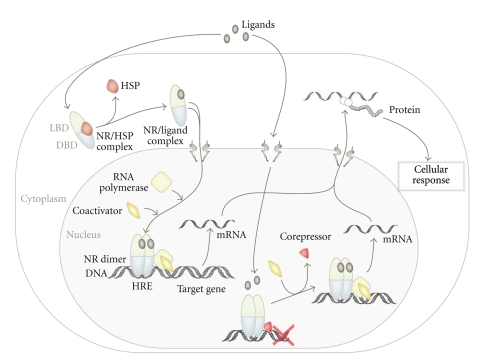
*Simplified model illustrating the two major modes of NR activation.* Natural or synthetic ligands diffuse through the cell membrane and bind to cytosolic or nuclear NRs. Ligand binding to cytoplasmic NRs triggers conformational changes resulting in dissociation of heat shock proteins (HSPs) and receptor dimerization, allowing active nuclear import and transactivation by binding to HREs. Other NRs are constitutively nuclear and complexed with corepressors in the absence of ligands. Ligand binding induces conformational changes resulting in the recruitment of coactivators to activate transcription of target genes.

**Figure 4:**
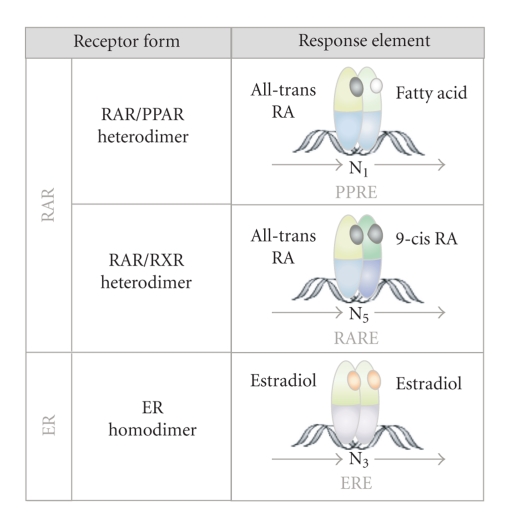
*DNA-binding modes of NRs implicated in HNSCC.* RAR can heterodimerize with PPARs, which can be activated by lipophilic ligands. Alternatively, RARs are able to heterodimerize with RXRs, which are activated by 9-*cis* RA. Such heterodimers can bind to specific half-site retinoic acid (RARE) or peroxisome proliferator response elements (PPREs) direct repeats in the DNA of target genes. Estradiol binding induces estrogen receptor homodimerization and binding to palindromic half-site estrogen response element (ERE) inverted repeats. N: Any nucleotide occurring within the specific response element.

In contrast to cell surface growth factor receptors, such as the epidermal growth factor receptor (EGFR), which activate genetic programs through complex intracellular signaling cascades, NRs are able to directly bind to specific DNA-sequences, so-called hormone response elements (HREs). Thus, NRs are composed of an N-terminal regulatory domain (activation function 1 = AF1), followed by a DNA-binding domain (DBD), a ligand-binding domain (LBD), and another C-terminal regulatory domain (activation function 2 = AF2) (Figure 2) [7, 8]. Despite their conserved structural organization, the biological functions of NRs are highly diverse. Nevertheless, two major modes of NR action can be assigned, depending on their intracellular steady-state localization in the absence of ligands (Figure 3). One group of NRs is confined to the cytoplasm within multiprotein-complexes in the absence of ligand. Upon ligand binding, they actively enter the nucleus and bind to HREs as homo- or heterodimers (Figure 4, details in [7, 8]). Other NRs already reside in the nucleus in a complex with corepressor proteins, while ligand binding triggers corepressor dissociation allowing the recruitment of coactivators [18, 19]. However, in order to fulfill multiple biological tasks minor to major deviations from these two modes of NR action exist [7, 8].

NRs are not only implicated in a broad spectrum of physiological processes but are associated with many human diseases including metabolic and cardiovascular disorders as well as cancer. Beside their proven clinical relevance for hormone regulated malignancies, there is rather limited information on their pathophysiological role as well as their prognostic and therapeutic potential for head and neck cancer [7, 8, 12, 20–22]. Most studies were investigating members of two classes of the NR superfamily, the thyroid hormone receptor-like and the estrogen receptor-like receptors (Table 1). Thus, we will focus on relevant members of these subfamilies, summarize their potential diagnostic/prognostic value, and discuss their therapeutic potential.

## 2. Thyroid Hormone Receptor-Like Receptors

### 2.1. Peroxisome Proliferator-Activated Receptors

Within the thyroid hormone receptor-like receptor subfamily, the peroxisome proliferator-activated receptors (PPARs) show the highest disease relevance for HNSCC. To date, three isoforms of the PPAR (*α*, *β*/*δ*, and *γ*) have been identified, all able to form heterodimers with retinoid X receptors (RXRs) (see [23, 24]). PPARs are expressed in different cell types and activate the transcription of several genes involved in a variety of biological processes, including lipid metabolism and insulin sensitivity (see [23, 24]). Furthermore, a role in limiting inflammation has also been reported [24, 25]. As tumor cell metabolism and inflammation appear to be critical for tumorigenesis and clinical outcome, NRs may thus directly or/and indirectly modulate malignancies [26, 27]. As such, PPAR*γ* is overexpressed in many epithelial malignancies [22, 28, 29] including oral squamous cell carcinoma [30].

In the absence of ligand, PPARs are complexed with corepressor proteins, thus acting as transcriptional repressors. Ligand binding induces conformational changes facilitating heterodimerization with RXR, thus leading to the attraction of transcriptional coactivators (Figures 3  and 4) (see [19, 24]). Natural and synthetic ligands for PPARs include lipophilic molecules such as fatty acids and eicosanoids as well as thiazolidinedione (TZD) drugs and derivates thereof (overview in [7, 24, 31]). PPAR*γ* ligands seem to exert their effects in a dosage-dependent manner [32], although the detailed mechanism is currently not yet resolved. The postulated cancer modulating mechanisms are diverse, including effects on Wnt signaling, inhibition of NF*κ*B, as well as the modulation of cell cycle regulators and pro- and antiapoptotic proteins, which have been linked with head and neck cancer (see [4, 23]).

**Clinical Aspects of Peroxisome Proliferator-Activated Receptors in HNSCC**

In HNSCC, overexpression on the protein level has been convincingly demonstrated for PPAR*β* and PPAR*γ* [12, 30, 33]. Agonist binding to PPAR*β* can induce cell differentiation, growth arrest, and apoptosis of cancer cells [34]. Additionally, such activating PPAR*β* ligands were shown to exert antiproliferative on human colon and breast cancers (details in [23, 24]) and were also suggested as potential chemopreventive agents for oral carcinogenesis [12, 35, 36]. Of note, since at least 1.6 million patients take antidiabetic drugs that function as PPAR*β* ligands, epidemiological data on their long-term effects on tumor prevention would therefore be of value to rationally design cancer chemoprevention trials.

Paradoxically, not only PPAR*γ* agonists are considered as potential therapeutic agents in cancer therapy but also antagonists were studied in this respect [30]. PPAR*γ* inhibition was shown to induce apoptosis and anoikis and inhibit tumor cell invasion in squamous cell carcinomas [30]. Moreover, the results of several studies indicated that the growth-inhibiting activity of PPAR*γ* ligands in OSCC may be PPAR*γ* independent [37]. Others showed that the observed effects were strongly dependent on PPAR*γ*-expression [12, 38] as well as on the type and concentration of the agonist [39].

In the majority of OSCC cases, PPAR*γ* mRNA could be detected by RT-PCR [37]. By immunohistochemical analysis of primary tumors, PPAR*γ* was often found in low-grade tumors, especially in tumor endothelium [38], and a favorable impact of PPAR*γ* expression on relapse-free survival of the patients could be demonstrated [40].

The beneficial effects of PPAR*γ* ligands on malignancies were tested in several clinical trials, but outcomes proved to be highly diverse. Some trials revealed 40% partial response rates, whereas others could not show any significant beneficial effect [41, 42].

Moreover, one may speculate that the tumor modulating effects of PPAR*γ* ligands are mediated indirectly by affecting the tumor microenvironment, such as cancer-associated fibroblasts or tumor endothelial cells [43]. In fact, PPAR*γ* ligands have been shown to affect endothelial cell proliferation and migration and hence to regulate angiogenesis [44]. Also hypoxia-induced angiogenesis appears to be affected by PPAR*γ* ligands in cancer therapy, even if the precise mechanisms still remain unclear [45]. As angiogenesis is a crucial aspect for tumor development, therapy resistance and metastasis and inhibition of angiogenesis may hence have contributed to the clinical benefit observed.

In sum, PPAR*γ* ligands appear to be of clinical benefit for the treatment of head and neck cancer, in particular for OSCC. Nevertheless, a more detailed molecular knowledge on PPAR*γ* biology is clearly required. Increasing knowledge about the mode of action, specificity, and dosage-dependence of PPAR agonistic and antagonistic ligands will hopefully allow a better modeling of PPAR receptor function and thus lead to a more effective design of combinatorial application schemes for cancer treatment and cancer prevention in the future.

### 2.2. Retinoid Acid Receptors

Another group of thyroid hormone receptor-like receptors implicated in HNSCC is the retinoid acid receptors (RARs). RARs are characterized by their activation via vitamin A derivatives. Upon activation, RARs are able to heterodimerize with retinoid X receptors (RXR) and to bind to specific hormone response elements (HREs), thereby modulating transcription of target genes (Figures 3  and 4) [8, 26, 46]. To date, a variety of coactivator- and corepressor-proteins have been identified, allowing the fine-tuning of target gene transcription, ranging from repression to full activation [18]. However, the molecular details are just beginning to be uncovered [8, 26, 46]. RAR activation often leads to differentiation [47], cell-cycle arrest [48], or apoptosis [49], culminating in the inhibiting of tumor growth. Hence, its ligand retinoid acid (RA) or derivates thereof are currently tested as therapeutic agents in several cancer types (Table 2) [50]. Paradoxically, in some malignancies RA rather promotes cell survival, which may be due to the ability of RA to also activate PPARs, and as a consequence expression of prosurvival genes is induced [46]. Schug et al. could also show that the channeling of RA between these two nuclear receptor heterodimers is mediated by the cytoplasmic RA transporters CRABP2 and FABP5 and thus is strongly depending on the FABP5/CRABP2 ratio [46]. Thus, the channeling of RA to different receptor heterodimers appears to be crucial for the regulation of cell-proliferation, positively or negatively affecting tumor growth [46]. Interestingly, both proteins were found differentially expressed in metastatic and HPV-associated HNSCC, but their biological and clinical effects remain to be investigated [51, 52].

An additional way of biological regulation is epigenetic modulation playing an important role in cancer development (reviewed in [53]). Gene silencing caused by aberrant hypermethylation of CpG islands has not only been detected in promoter regions of several tumor suppressor genes [53], but several studies show hypermethylation of the RAR*β* promoter in colon, breast, and lung cancers [54, 55]. In head and neck carcinogenesis, hypermethylation of the RAR*β* promoter was found to be indeed associated with RAR*β* downregulation and hence appears to be biologically relevant [56].

**Clinical Aspects of Retinoid Acid Receptors in HNSCC**

As outlined above, a rationale for the use of retinoids in chemoprevention and cancer therapy was provided experimentally by different cellular [57] and animal models [58]. Moreover, this strategy was supported by epidemiological data as well as by clinical trial outcomes [26, 59–61]. Several clinical chemoprevention trials including patients with increased risk for developing cancer have shown that treatment with retinoids resulted in the suppression of precancerous lesions (see [26, 60]). Also, certain retinoids inhibited the development of second primary tumors in patients who had been previously treated for an early-stage cancer but remained at high risk to relapse ([26, 60] and references within). However, other studies using isotretinoin or other retinoids (e.g., retinyl palmitate) did not observe any benefit in second primary tumor development, recurrence, or mortality of HNSCC or lung cancer [26, 62]. Current trials (Table 2) are therefore aiming to resolve these controversies by recruiting appropriate study populations as well as by the use of novel drugs and improved treatment protocols.

Reduced RAR*β* mRNA levels have been observed not only in several malignant tumors ([26, 56] and references therein) but also in premalignant oral lesions ([63] and references within). Unfortunately, until recently no antibodies convincingly detecting RAR*β* were available. Thus, most of the studies demonstrating RAR*β* downregulation were based on in situ hybridization and could therefore only show a decrease in the amount of mRNA. Ralhan et al. were the first to demonstrate decreased expression of the RXR*α* and RAR*α*/*β*/*γ* on protein level correlating with different stages of OSCC development and progression [64]. The molecular mechanism leading to downregulation or loss of RAR*β* is poorly understood, but it was suggested that expression of RAR*β* could depend on the intracellular level of retinoids [26]. Several studies demonstrated a decrease in the amount of RAR*β* during vitamin A deficiency as well as its upregulation by RA. Additionally, there is evidence that retinoic acid induces the expression of RAR*β* mRNA in certain cell lines, but not in the malignant counterparts of these cells. Thus, transformed cells may have developed an aberrant response to retinoic acid due to the deregulated expression of coactivator/repressor proteins [26]. Ralhan et al. found a significant association between the increase in RAR*β* mRNA levels and clinical responses of premalignant oral lesions to isotretinoin [65, 66]. Hence, RAR*β* indeed seems to contribute to the suppression of the premalignant phenotype and malignancy and may be causally linked to the clinical outcome in chemoprevention trials with retinoids [26, 67]. If so, RAR*β* may indeed serve as a useful diagnostic marker in retinoid trials (Table 2) for the prevention of oral carcinogenesis [68]. RAR modulation by its agonist ligand all-*trans* retinoic acid (ATRA) represents a successful example of how targeting of an NR contributes to an impressive clinical benefit in liquid tumors ([26] and references therein). Lessons learned from these studies clearly show that the therapeutic benefit could be further enhanced by combining ATRA with chromatin modulating agents, such as histone deacetylase inhibitors [69]. Nevertheless, the design of receptor specific drugs as well as an in depth understanding of the molecular regulation of RAR biology is required in order to fully exploit its therapeutic benefit and minimize potential side-effects in the area of head and neck cancer [7, 26].

## 3. Estrogen Receptor-Like Receptors

This subfamily is composed of the estrogen receptors (ER*α* and ER*β*), the estrogen-related receptor, and the 3-ketosteroid receptors [10]. 

Besides the estrogen receptors themselves, many of the genes regulated by the ER/estrogen-axis are critical for cell proliferation, inhibition of apoptosis, stimulation of invasion and metastasis, as well as for the promotion of angiogenesis (see [10, 11] and references within). Since these processes clearly state hallmarks of cancer cells, it is well accepted that ERs are implicated in various cancer types [9, 21]. Sex hormone receptors are expressed not only in sexual organs but, amongst others, also in the vascular epithelium [70], the lung epithelium [71], and the larynx [72]. The expression of sex hormone receptors could also be demonstrated in HNSCC by several studies [12, 13]. Both ER isoforms as well as the progesterone receptor (PR) were detectable in cancer cells of the oral cavity, the salivary gland, and in laryngeal/hypopharyngeal cancers, whereas the tumor stroma was mostly negative [12, 13]. Expression of ER*α* inversely correlated with that of ER*β* in esophageal carcinoma, and a correlation of ER*β* levels with tumor dedifferentiation and staging was suggested [73, 74].

**Clinical Aspects of Estrogen Receptors in HNSCC**

Considering the impressive benefit of endocrine therapy in breast cancer, targeting sex steroid hormone receptor as a potential therapeutic strategy is also discussed for HNSCC [12, 75]. Currently, two main strategies are pursued in endocrine therapy of ER-positive tumors. One is based on steroidal antiestrogens like tamoxifen, which bind to the ER, block its function, and ultimately induce receptor degradation [8, 11]. The other is based on aromatase inhibitors and luteinizing hormone-releasing hormone agonists, which reduce the level of circulating estrogen, thereby inhibiting ER activation by depriving the receptor of its ligand [11]. Tamoxifen was already shown to inhibit proliferation and invasion of HNSCC cell lines, resulting in apoptosis, which could be further enhanced upon combination with cisplatin [76–78]. Thus, a therapeutic role of antiestrogens or aromatase inhibitors in the clinical management of HNSCC is currently under investigation, and the results of just completed clinical trials (Table 2) are eagerly awaited.

However, the precise molecular roles and impact of estrogen receptor-like receptors for the onset and/or progression of head and neck cancer remain to be clarified. This knowledge will be required, in order to rationally decide whether to further investigate the potential of modern endocrine therapy also for this tumor entity.

## 4. Conclusion

NRs are associated with head and neck cancer and hence seem to be at least partially amenable for prevention and/or treatment strategies. So far, three NR groups have mainly been linked with HNSCC, the retinoic acid and the peroxisome proliferator-activated and the estrogen receptors. Also, target genes activated by these NR subfamilies (Table 3) have been implicated as key elements in the molecular circuits involved in head and neck cancer development and progression. Reports on other members of the NR superfamiliy are rather scarce for this tumor entity, suggesting that they have not been investigated so far. Taking the thyroid hormone receptor as an example, many studies on its relevance for various malignancies have been conducted, whereas its role in head and neck cancer, including even thyroid carcinomas, has not been analyzed in detail [79]. Likewise, data on the cancer-related biological functions of orphan NRs are still missing for this tumor entity [7, 8]. As now cancer cell metabolism is beginning to be considered as “cancer's Achilles' heel”, it may be conceivable to speculate that molecules present in diet, tobacco, or beetle nut might deregulate the cell's metabolism by affecting NRs and as such contribute to head and neck carcinogenesis [27, 80]. Of note, the development of novel NR ligands with improved specificity and activity is currently intensively pursued in the area of metabolic diseases (see [7, 8, 81]). Hence, an interdisciplinary exploitation of the existing knowledge of NR pharmacobiology may result in novel HNSCC treatment approaches.

In sum, keeping in mind the enormous success of NR targeting therapeutics in several malignancies, a systematic investigation of NR biology as well as of its clinical relevance is highly desirable also for head and neck cancer. Together with the outcomes of current clinical trials (Table 2), such improved knowledge will hopefully result in strategies with improved benefit for the patient.

## Figures and Tables

**Table 1:** Current classification of the NR superfamily into subfamilies according to sequence homology. Trivial abbreviations are given in brackets. NRs implicated in head and neck tumorigenesis are given in bold; asterisks indicate orphan receptors.

Subfamily	Full name	Subfamily members (trivial abbreviation)
Subfamily 1	Thyroid hormone receptor-like receptors	

Subgroups	**Peroxisome proliferator-activated receptors**	**Peroxisome proliferator-activated receptor (PPAR) ** ***α*, *β*/*δ*, *γ***
	**Retinoic acid receptors**	**Retinoic acid receptor (RAR) ** ***α*, *β*, *γ***
	Retinoic acid receptor-related orphan receptors	Retinoic acid receptor-related orphan receptor (ROR) *α*, *β*, *γ*
	Rev-ErbA*	Rev-ErbA (EAR1) *α*, *β*
	Thyroid hormone receptors	Thyroid hormone receptor (TR) *α*, *β*
	Liver X receptor-like receptors*	Liver X receptor (LXR) *α*, *β*; Farnesoid X receptor (FXR)
	Vitamin D receptor-like receptors	Vitamin D receptor (VDR); Pregnane X receptor (PXR); Constitutive androstane receptor (CAR)

Subfamily 2	Retinoid X receptor-like receptors	

Subgroups	Hepatocyte nuclear factor-4	Hepatocyte nuclear factor-4 (HNF-4) *α*, *γ*
	Retinoid X receptors	Retinoid X receptor (RXR) *α*, *β*, *γ*
	Testicular receptors*	Testicular receptor 2, 4 (TR2/4)
	Tailless-like receptors*	Human homologue of the Drosophila tailless gene (TLX); Photoreceptor cell-specific nuclear receptor (PNR)
	Chicken ovalbumin upstream promoter-transcription factor-like receptors*	Chicken ovalbumin upstream promoter-transcription factor (COUP-TF) I, II; V-erbA-related (EAR2)

Subfamily 3	Estrogen receptor-like receptors	

Subgroups	**Estrogen receptors**	**Estrogen receptor (ER) ** ***α*, *β***
	Estrogen related receptors*	Estrogen-related receptor (ERR) *α*, *β*, *γ*
	**3-Ketosteroid receptors**	**Androgen receptor (AR); Progesterone receptor (PR);**Glucocorticoid receptor (GR); Mineralocorticoid receptor (MR);

Subfamily 4	Nerve growth factor IB-like receptors	

	Nerve Growth factor IB/ Nuclear receptor related/ Neuron-derived orphan receptor*	Nerve Growth factor IB (NGF-IB); Nuclear receptor related 1 (NURR1); Neuron-derived orphan receptor 1 (NOR1)

Subfamily 5	Steroidogenic factor-like receptors	

	Steroidogenic factor/Liver receptor homolog*	Steroidogenic factor 1 (SF1); Liver receptor homologue-1 (LHR1)

Subfamily 6	Germ cell nuclear factor-like receptors	

	Germ cell nuclear factor*	Germ cell nuclear factor (GCNF)

Subfamily 0	Miscellaneous receptors	

	Dosage-sensitive sex reversal, adrenal hypoplasia critical region/Small heterodimer partner*	Dosage-sensitive sex reversal, adrenal hypoplasia critical region, on chromosome X, gene 1 (DAX); Small heterodimer partner (SHP)

**Table 2:** Overview of current clinical trials in the field of HNSCC targeting NRs. The NCI protocol ID is given in bold (for further details see: http://www.cancer.gov/CLINICALTRIALS).

NR	Clinical trial / identifier	Drug	Tumor entity	Phase
PPAR	Pioglitazone in Preventing Head and Neck Cancer in Patients With Oral Leukoplakia/NCT00099021	Pioglitazone	Head and Neck Cancer *Precancerous/Nonmalignant Condition *	Phase II ongoing
	Rosiglitazone in Preventing Oral Cancer in Patients With Oral Leukoplakia/NCT00369174	Rosiglitazone	Head and Neck Cancer * Precancerous/Nonmalignant Condition *	Phase II completed

RAR	Chemoprevention Study of Oral Cavity Squamous Cell Carcinoma/NCT00201279	13-cis Retinoic acid	Oral Cavity Squamous Cell Carcinoma	Phase III completed
	Isotretinoin Plus Interferon in Treating Patients With Recurrent Cancer/NCT00002506	Isotretinoin (combined with Interferon a)	Head and Neck Cancer Esophageal Cancer	Phase II ongoing
	Isotretinoin, Interferon Alpha, and Vitamin E in Treating Patients With Stage III or Stage IV Head and Neck Cancer/NCT00054561	Isotretinoin (combined with Interferon a and Vitamin E)	Head and Neck Cancer	Phase III completed

ER	Combination Chemotherapy and Tamoxifen in Treating Patients With Solid Tumors/NCT00002608	Tamoxifen (combined with Cisplatin and Doxorubicin)	Head and Neck Cancer	Phase II completed

**Table 3:** Nuclear receptor target genes playing pivotal roles in diverse biological processes and cellular homeostasis were described to be differentially expressed in head and neck cancer.

NR	Target gene	Function	Reference - target gene	Reference - head and neck
PPAR	**G0S2**	Cell cycle	Zandbergen et al. *Biochem J* 2005	Tokumaru et al. *Cancer Res* 2004
	**PDK1**	Energy homeostasis	Degenhardt et al. *J Mol Biol* 2007	Wigfield et al. *Br J Cancer* 2008

RAR	**p21WAF1/CIP1**	Cell cycle	Liu et al. *J Biol Chem* 1996	Kapranos et al. *Anticancer Res* 2001
	**BIRC5/surviving**	Apoptosis	Pratt et al. *J Cell Biochem* 2003	Engels et al. *J Pathol* 2007
	**C/EBP*ε***	Transcription factor	Schwarz et al. *Mol Cell Biol* 1997	Bennett et al. *Cancer Res* 2007
	**CRABP**	Carrier protein	Nezzar et al. *Mol Vis* 2007	Won et al. *Metabolism* 2004
	**cyclins**, **CDK**	Cell cycle	Bour et al. *Trends Cell Biol* 2007	Jeannon et al. *Clin Otolaryngol Allied Sci* 1998

**ER**	**c-Myc**	Transcription factor	Markaverich et al. *Steroids* 2006	Pries et al. *Int J Mol Med* 2008
	**Cyclins**	Cell cycle	Eeckhoute et al. *Genes Dev* 2006	Nakashima et al. *Eur Arch Otorhinolaryngol* 2005 Lotayef et al. *Br J Cancer* 2000
	**CRABP**	Carrier protein	Li et al. *J Biol Chem* 2003	Vo et al. *Anticancer Res* 1998
	**CXCl12/SDF-1**	Chemokine/ligand	Hall et al. *Mol Endocrinol* 2003	Rehman et al. *J Biol Chem* 2008
	**cathepsin D**	Protease	Bretschneider et al. *Mol Oncol* 2008	Strojan et al. *Anticancer Res* 2000
